# Discriminant Function Analysis of Foramen Magnum Variables in South Indian Population: A Study of Computerised Tomographic Images

**DOI:** 10.1155/2018/2056291

**Published:** 2018-09-26

**Authors:** S. P. Vinutha, V. Suresh, R. Shubha

**Affiliations:** ^1^Assistant Professor, Department of Anatomy, JSS Medical College, Mysore, Karnataka, India; ^2^Associate Professor, Department of Radiodiagnosis, Kempegowda Institute of Medical Sciences, Bangalore, Karnataka, India; ^3^Professor, Department of Anatomy, Kempegowda Institute of Medical Sciences, Bangalore, Karnataka, India

## Abstract

**Background and Objectives:**

The foramen magnum as a transition area between skull and spine plays an important role as a landmark, because of its close relationship to vital structures such as the brain and spinal cord. Configuration and size of the foramen magnum play an important role for assessing craniovertebral relations. The objectives of the present study are to find out shape and dimensions of foramen magnum in cranial CT scans. All the variables were evaluated by using discriminant function analysis.

**Materials and Methods:**

The study sample comprised 200 CT scans (110 males and 90 females) of south Indian origin. The shapes of the foramen magnum were classified into 8 types: oval, egg, round, tetragonal, pentagonal, hexagonal, irregular (A), and irregular (B). The parameters were noted meticulously and the statistical analysis for sex comparison was made by Student's* t*-test and was considered significant whenever* P*≤0.05. To determine the relationships between the studied parameters, Pearson correlation coefficients were calculated.

**Results:**

The foramen magnum was classified into 8 types based on shape. Commonest was oval and pentagonal was the least common type. The anteroposterior diameter, transverse diameter, circumference, and area were significantly greater in males than in females (*P*<0.001). The mean FM index of male CT scans was 85.01 ± 8.67, whereas in case of female CT scans, it was 83.17 ± 7.78. With all the variables in consideration, overall 65% of cranial CT scans were sexed correctly by using multivariate analysis.

**Conclusion:**

Several anatomic parameters such as shape and dimensions of FM should be taken into consideration during surgery involving the craniovertebral junction. Also these can be used during forensic and anthropological investigation of unknown individuals for determining gender, ethnicity, and so on. The multivariate analysis is by far the best method for determination of sex of cranium with available resources.

## 1. Introduction

Foramen magnum (FM) is the largest foramen in the skull. It lies in an anteromedian position and leads into the posterior cranial fossa. It is oval, wider behind, with its greatest diameter being anteroposterior. It contains the lower end of the medulla oblongata, meninges, vertebral arteries, and the spinal accessory nerve [1].

The shape of foramen magnum varies, commonest being oval. The importance of variations in shape is due to its effects on the vital structures passing through it and also plays an important role in various surgical approaches. Dimensions of the foramen magnum have clinical importance because the vital structures that pass through it may suffer compression. It has also been observed that longer anteroposterior dimension of foramen magnum permitted greater contralateral surgical exposure for condylar resection. The anatomic and radiologic values of the foramen magnum have been the objective of several studies. Although the anatomic values obtained by different authors are nearly the same, this does not happen with radiologic values [2].

Measurements of various bones are often used during forensic and anthropological investigations of unknown individuals for estimation of age, gender, stature, and ethnicity. Base of the skull is covered by large mass of soft tissue which helps to protect the foramen magnum. So in cases of severe trauma, fire, explosions, and so on, an intact foramen magnum morphometry helps in determining the gender and consequent identification of a person. Hence, morphometry of foramen magnum becomes important [3].

Foramen magnum dimensions are specific for a particular population and become low, when applied to populations with a large ethnic mix. From qualitative and quantitative point of view, the features and morphometry of foramen magnum and occipital bone, when used in approximation, are good indicators for the diagnosis of sex [4].

Configuration and size of the foramen magnum play an important role in the pathophysiology of various disorders of the craniovertebral junction. Thus, a fundamental knowledge of normal anatomy and basic craniometric measurements for assessing craniovertebral relations is important to the clinician who diagnoses disorders affecting this region or the surgeon who operates on this anatomy [9].

Not many studies have been done pertaining to morphometry and sexual dimorphism in foramen magnum in south Indian population. Hence, this study of discriminant function analysis of foramen magnum variables involving cranial CT scans becomes essential.

This study was undertaken to evaluate the following parameters related to foramen magnum in cranial CT images:

1.
  - Shape
  - Anteroposterior diameter
  - Transverse diameter
  - Circumference
  - Area
  - Foramen magnum index
2. Multivariate discriminant function analysis was used to analyze sex differences within the skulls.

## 2. Materials and Methods

A retrospective radiological study was done on 200 (110 males and 90 females) cranial CT scans of known gender available in the Department of Radiodiagnosis, Kempegowda Institute of Medical Sciences, Bangalore, Karnataka, India. The study was approved by the institutional ethics committee of Kempegowda Institute of Medical Sciences, Bangalore.

**Inclusion criteria:** Adult cranial CT scans with complete foramen magnum.

**Exclusion criteria:** Cranial CT scans with damaged foramen magnum and when associated with pathological conditions.

In cranial CT scans, from continuous 5 mm thick slices and parallel to the orbitomeatal line, the best image of the FM was selected. By visual examination of the images, variations in the shape of the foramen magnum were noted. The shapes of the FM were classified into 8 types: oval, egg, round, tetragonal, pentagonal, hexagonal, irregular (A), and irregular (B). The shape irregular (A) corresponds to combination of two different semicircles and the shape irregular (B) could not be classified into any of the shapes abovementioned as shown in Figures 2(g) and 2(h).

**Figure 1:**
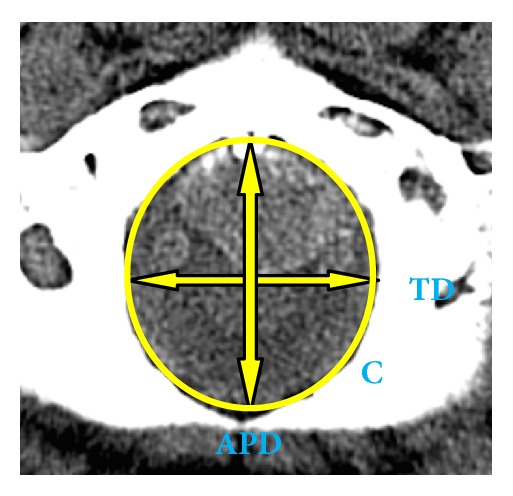
Cranial base demonstrating the foramen magnum measurements.

The maximum anteroposterior diameter (**APD**) was measured from the basion (the midpoint of the anterior margin of the FM) to the opisthion (the midpoint of the posterior margin of the FM). The maximum transverse diameter (**TD**) was measured between the lateral margins of the FM at the point of greatest lateral curvature. The APD, TD, and circumference (**C**) of FM were measured by a millimetric sliding caliper and expressed as millimetres as shown in Figure 1. Area of the FM was automatically given in the CT images as millimetres square.

The data obtained were tabulated and analysed statistically by computing descriptive statistics like mean, standard deviation, range, and percentages. The statistical analysis for sex comparison was made by Student's* t*-test. The results were considered significant when* P* ≤ 0.05 and considered highly significant when* P* ≤ 0.001. To determine the relationships between the studied parameters, Pearson correlation coefficients were calculated.

Foramen magnum index was calculated using the formula:

(1) $$\begin{array}{c} \frac{Transverse\;\;diameter}{Anteroposterior\;\;diameter}\times 100 \end{array}$$

The foramen magnum index was evaluated according to the Martin and Saller classification [9].

- Narrow: x - ≤81.9
- Medium: 82.0 - 85.9
- Large: ≥86.0 - x.

Wilk's lambda is used in an ANOVA (F) test of mean differences in discriminant analysis, such that the smaller the lambda for an independent variable, the more that variable contributes to the discriminant function. Lambda varies from 0 to 1; the value closer to zero discriminates more between males and females. The F test of Wilk's lambda shows which variable contributions are significant. The principle of multivariate linear discriminant function is that measured variables are taken as independent variables whereas sex is a dependent variable. The sectioning point was created by using the mean male and female discriminant scores known as the group centroids [10].

Data was analyzed by SPSS programme version 22.0 and coefficients were obtained. The formula was D = b0 + b1X1 + b2 X2 + b3 X3 + b4 X4 (b0 is constant, b1–b4 are coefficients, and X1–X4 are variable of parameters). In the above formula mean values of respective male and female variables were used and the functional score was designated as D_m_ for male and D_f_ for female, respectively. Sectioning point (D_0_) was obtained by putting the average value of mean of male and female variables in place of X [10].

## 3. Results

**Figure 2:**
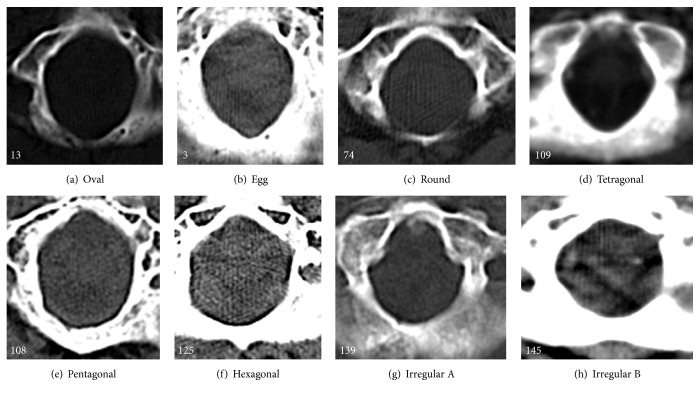
Different shapes of foramen magnum as studied in cranial CT scans.

Oval shape was the most common type and pentagonal was the least common type in cranial CT scans of both genders.  Table 1 shows the number and percentages of different types of FM shape.  Figure 2 shows the different shapes of foramen magnum as studied in cranial CT scans.

Table 2 shows the results of the study in 200 CT scans that the APD, TD, circumference, and area were significantly greater in males than in females (*P* < 0.001).

The results showed the** medium** type of** FM index **according to Martin and Saller classification. The mean FM index was 84.27 ± 8.35 (combined). Even though the FM index was higher in male CT scans (85.01 ± 8.67) when compared to female CT scans (83.17 ± 7.78), the difference was not statistically significant (*P*>0.05).

## 4. Discriminant Function Analysis

We notice that each variable is a significant predictor in sexing a given sample (*P*<0.001). FM transverse diameter (Wilk's lambda = 0.841, F = 37.553) was the best discriminator followed by FM circumference (Wilk's lambda = 0.861, F = 31.848) (Table 3).  Table 4 shows the unstandardized, standardized, and structure coefficients in the original samples of CT scans.  Table 5 shows the linear discriminant function for CT scans.

It was observed that 70 male cranial CT scans out of 110 were sexed correctly and 60 female cranial CT scans out of 90 were sexed correctly. Overall 130 out of 200 cranial CT scans were sexed correctly. So the percentage of crania which were sexed correctly for male was 63.6% and for female it was 66.6%. With all the variables in consideration, overall 65% of cranial CT scans were sexed correctly.

Pearson's correlation equation was applied for all FM measurements in male CT scans. There was a significant correlation among all the parameters studied (*P*<0.001). The strongest positive correlation was observed between circumference and area (r = 0.930). The weakest positive correlation was observed between anteroposterior diameter and transverse diameter (r = 0.472) (Table 6).

Pearson's correlation equation was applied for all FM measurements in female CT scans. There was a significant correlation among all the parameters studied (*P*<0.001). The strongest positive correlation was observed between circumference and area (r = 0.930). The weakest positive correlation was observed between anteroposterior diameter and transverse diameter (r = 0.582) (Table 7).

## 5. Discussion

Development of a particular shape of the FM is explained on the basis of the embryologic data. It may be caused due to ossification of primordial cranial residues, which join the endochondral ossification points in different locations, resulting in various shapes [9]. Irregular shape of FM is accentuated by the developmental anomalies of the bone and soft tissues at the craniovertebral junction [11].

The degree of expression of sexual dimorphism within the FM dimensions can be described by its development. Compared to many other skeletal elements, the foramen magnum reaches its adult size relatively early in childhood and is not likely to respond to significant secondary sexual changes. No muscles act upon the shape and size of the FM; its prime function is to accommodate the passage of structures into and out of the cranial base region particularly the medulla oblongata, which occupies the greatest proportion of the foramina space. As the nervous system is the most precocious of all body systems, it reaches maturity at a very young age and therefore has no requirement to increase in size. This is evidenced by the completion of fusion of the different elements of the occipital bone by 5–7 years of age [12].

In the present study, we have classified the foramen magnum into 8 shapes based on the study by Murshed et al. [2]. No literature was available regarding the gender differences in FM in CT scans based on shape. Oval shape (36.5%) was the most common type in the present study. Garcia et al. [5] have found 45% of oval shape FM in their study, which is higher than our findings. Murshed et al. [2] have found 8.1% of oval shape FM, which is much lower than our values. These differences may be because Murshed et al. [2] studied Turkish population and Garcia et al. [5] studied South American population. Also since Garcia et al. have classified the FM shapes into 7 types only, the oval shape might have included the irregular (B) shape also.

Murshed et al. [2] have found 9.09% of irregular (B) shape in their study, which is almost similar to our results. However, Garcia et al. [5] have not classified the irregular shape further into irregular (A) and irregular (B). The authors concluded that when shape is considered, it is dependent on the observer experience and also replicability is not high. Hence, variations between different studies may exist.

Murshed et al. [2] have found in their study that round shape (21.8%) FM was the most common type and egg shape (6.3%) was the least common type. This difference may be due to variation in the classification of oval and round shapes because in their study, percentage of oval shape FM has decreased and there is a reciprocal increase in the percentage of round shape FM.

The shape of the foramen magnum can be calculated by using foramen magnum index. Muthukumar et al. have considered FM to be oval when the FM index was ≥ 1.2, and they considered the rest (FM index < 1.2) as circular. A similar sized lesion situated anterior to the brainstem will require more extensive bone removal in a person with an ovoid FM than in a person with a circular FM. In 20% of the skulls, the occipital condyle protruded significantly into the foramen magnum. So, a patient with a round foramen magnum without significant protrusion of the occipital condyles into the foramen magnum will require less bony resection than a patient with an ovoid foramen magnum with medially protuberant and sagittally inclined occipital condyles; however both patients have similar lesions [13].

Tables 8 and 9 shows the results of various dimensions of FM (measurable parameters) in the present study were compared with the results of previous studies. This is more important because metric approach is more objective and less dependent on observer experience. So, interobserver variations might be less. Its replicability is high and is also more responsive to statistical analysis. This helps comparisons between samples and also between studies.

Other studies have reported slightly higher mean values of FM when compared to the present study. This may be due to racial differences as the study population is different or may be due to methodological differences and also due to variations in the sample size.

Only one study by Uthman et al. [6] has reported slightly lower mean values, when compared to our study. This difference may be because their sample size is small (n = 88) and also they have used reformatted axial sections using helical CT scan. Other 4 studies have reported slightly higher mean values because Garcia et al. [5] have studied South American population and Murshed et al. [2], Erdil et al. [7], and Uysal et al. [8] have studied Turkish population. The differences may also be due to the different radiologic techniques used by the different authors [2].

The minor controversies may have resulted from differences of the selected population, used parameters, methodological difference of data assessment, ignoring sex differences, and results of the studies based on dry skulls or cadaveric material. The authors concluded that morphometric analysis of foramen magnum shows variations so that interested region should be known in detail. Documenting morphometric values of foramen magnum on different populations is very important [7].

Many authors have reported the usefulness of the FM in gender estimation. But the data compiled for a certain population cannot be employed for determining sex in another population [14].

As quoted by the authors, Giles and Elliot have stated that “next to the pelvis, the skull is the most easily sexed portion of the skeleton.” These authors have developed a discriminant function technique that utilized cranial measurements in intact skulls, with 82–89% accuracy in predicting the sex. Holland suggests that the measurements of the region of occipital condyles and foramen magnum are useful for sex determination with accuracy of 70–90%.

The factors reducing the author's accuracy rates are probable methodological differences, as Holland made manual measurements in human skulls compared to author's measurements in a living human population by 3D CT. The authors concluded that radiological support should provide easier measurements improving the reliability of statistical analysis. CT/3D CT can be accurately used in further investigations to provide basis for anthropometric and forensic issues [8].

In the present study, 63% of the crania were classified correctly in overall CT scans. Our findings are lesser than those of Uthman et al. [6] and Uysal et al. [8]. The difference noted may be due to the number of variables studied while doing multivariate analysis. In case of present study it was 4 variables, and in case of study by Uthman et al. [6] it was 6 variables, whereas Uysal et al. [8] studied 7 variables. Hence, although our sample size is much larger than the other two, since we have studied only 4 variables, our percentage is lesser than the other two.

The foramen magnum as a transition zone between spine and skull plays an important role as a landmark because of its close relationship to important structures such as the brain and spinal cord [15].

The FM carries a great importance in the lateral approaches due to its localization in skull base region. The anterolateral aspect of the FM is one of the deepest and most complex areas of the skull base. Exposure of intra- or extradural lesions involving the FM and brainstem poses a significant surgical challenge for neurosurgeons since the foramen is covered by thick bony prominences where many nerves or vascular structures pass through [16].

There are several developmental variations in the region of the craniocervical junction. Some variations are minor anatomic abnormalities, but they can cause severe diagnostic problems. A reliable and exact radiologic diagnosis requires detailed knowledge of the morphologic features of the variations and the appearance of their characteristic features in the common radiologic procedures [17].

The craniovertebral junction (CVJ) consists of the foramen magnum and its bony frame, the atlas, and the axis [18]. Radiologic evaluation of the craniovertebral junction requires identification of few anatomic structures, knowledge of some basic osseous relationships, and a few craniometric measurements [19].

Anomalies and malformations of the occipital sclerotomes result in irregular geometry of the FM and related structures. Shape and size of the foramen are critical parameters for the manifestation of clinical signs and symptoms in craniocervical pathology. These include motor myelopathy, sensory abnormalities, brainstem and lower cranial nerve dysfunctions, and signs and symptoms referable to vascular compromise. Diseases associated with anomalies of the FM include occipital vertebra, basilar invagination, condylar hypoplasia, and atlas assimilation. Interestingly, one report found that the persistence of the spheno-occipital synchondrosis, aggravated by the coexistence of basilar invagination, resulted in stenosis at the foramen magnum [20].

## 6. Conclusion

It can be concluded that the several anatomic parameters such as shape and dimensions of FM should be taken into consideration during surgery involving the craniovertebral junction. Also these can be used during forensic and anthropological investigation of unknown individuals for determining gender, ethnicity, and so on. The multivariate analysis is by far the best method for determination of sex of cranium with available resources.

## Figures and Tables

**Table 1:** The number and percentages of various shapes of FM in CT scans.

**Types of shapes**	**Male CT scans**		**Female CT scans**		**Combined CT scans**	
	**No.**	%	**No.**	%	**No.**	%
Oval	**38**	**35.0**	**35**	**39.0**	**73**	**36.5**
Egg	10	9.0	8	8.9	18	9
Round	11	10.0	6	6.6	17	8.5
Tetragonal	8	7.2	6	6.6	14	7
Pentagonal	**5**	**4.5**	**4**	**4.5**	**09**	**4.5**
Hexagonal	16	14.5	14	15.5	30	15
Irregular (A)	12	10.8	9	10.0	21	10.5
Irregular (B)	10	9.0	8	8.9	18	9
Total	**110**	**100**	**90**	**100**	**200**	**100**

**Table 2:** Descriptive statistics of measured parameters of FM in both genders of CT scans.

**Variable **	**Gender**	**Mean**	**SD**	**SE of mean**	**Min**	**Max**	**t**	***P* value**
Anteroposterior diameter (mm)	Male	35.96	3.75	0.34	28.30	45.60	4.053	**<0.001** **∗**
	Female	33.83	3.51	0.39	24.70	41.40		
Transverse diameter (mm)	Male	30.38	2.84	0.26	23.30	40.30	6.128	**<0.001** **∗**
	Female	27.98	2.53	0.28	23.00	34.30		
Circumference (mm)	Male	124.87	10.49	0.96	105.00	156.00	5.643	**<0.001** **∗**
	Female	116.60	9.67	1.07	97.00	137.00		
Area (mm^2^)	Male	799.29	134.05	12.29	537.05	1198.28	5.536	**<0.001** **∗**
	Female	697.77	116.63	12.96	451.41	991.08		

**Table 3:** Discriminant function analysis using FM measurements in CT scans.

**Variable**	**Male**		**Female**		**Wilk's** ***λ***	**F**	***P* value**
	**Mean**	**SD**	**Mean**	**SD**			
APD (mm)	35.96	3.75	33.83	3.51	0.923	16.425	**<0.001** **∗**
TD (mm)	30.38	2.84	27.98	2.53	0.841	37.553	**<0.001** **∗**
Circumference (mm)	124.87	10.49	116.60	9.67	0.861	31.848	**<0.001** **∗**
Area (mm^2^)	799.29	134.05	697.77	116.63	0.866	30.649	**<0.001** **∗**

*∗* denotes a significant factor.

**Table 4:** Standardized and unstandardized coefficients in original samples of CT scans.

**Variable**	**Unstandardized coefficients**	**Standardized coefficients**	**Structure coefficients**
Anteroposterior diameter	-0.049	-0.179	0.954
Transverse diameter	0.257	0.698	0.878
Circumference	0.078	0.792	0.861
Area	-0.002	-0.289	0.631
Constant	-13.585	-* *-* *-	-* *-* *-

**Table 5:** Linear discriminant function for CT scans.

**Variable**	**Male**	**Female**
Constant	-297.702	-285.207
Anteroposterior diameter	7.539	7.584
Transverse diameter	12.586	12.348
Circumference	3.398	3.326
Area	-0.605	-0.603
Group centroids	0.375	-0.551

**Table 6:** Correlation among measured variables of FM in male CT scans.

**Correlations**		**APD**	**TD**	**Circumference**	**Area**
**APD**	r	1	**0.472**	0.879	0.842
	*P* value	-* *-* *-	<0.001*∗*	<0.001*∗*	<0.001*∗*
**TD**	r	**0.472**	1	0.710	0.800
	*P* value	<0.001*∗*	-* *-* *-	<0.001*∗*	<0.001*∗*
**Circumference**	r	0.879	0.710	1	**0.930**
	*P* value	<0.001*∗*	<0.001*∗*	-* *-* *-	<0.001*∗*
**Area**	r	0.842	0.800	**0.930**	1
	*P* value	<0.001*∗*	<0.001*∗*	<0.001*∗*	-* *-* *-

*∗* denotes significant correlation.

**Table 7:** Correlation among measured variables of FM in female CT scans.

**Correlations**		**APD**	**TD**	**Circumference**	**Area**
**APD**	r	1	**0.582**	0.886	0.872
	*P* value	-* *-* *-	<0.001*∗*	<0.001*∗*	<0.001*∗*
**TD**	r	**0.582**	1	0.775	0.831
	*P* value	<0.001*∗*	-* *-* *-	<0.001*∗*	<0.001*∗*
**Circumference**	r	0.886	0.775	1	**0.930**
	*P* value	<0.001*∗*	<0.001*∗*	-* *-* *-	<0.001*∗*
**Area**	r	0.872	0.831	**0.930**	1
	*P* value	<0.001*∗*	<0.001*∗*	<0.001*∗*	-* *-* *-

*∗* denotes significant correlation.

**Table 8:** Comparison of foramen magnum variables in male CT scans with previous studies.

**Authors name **	**Sample size**	**Target population**	**APD**	**TD**	**Circumference**	**Area**
Murshed et al. [2] 2003	110	Turkish population	37.2 ± 3.43	31.6 ± 2.99	-	931.7 ± 144.29
Garcia et al. [5] 2011	100	South American	37.4 ± 3.3	31.9 ± 2.6	-	877 ± 125
Uthman et al. [6] 2012	88	Iraqi population	34.9 ± 2	29.5 ± 2.5	99.3 ± 6.2	765.2 ± 98
Erdil et al. [7] 2010	54	Turkish population	36.95 ± 4.01	30.75 ± 2.81	-	-
Uysal et al. [8] 2005	100	Turkish population	37.08 ± 1.93	30.83 ± 2.04	-	-
**Present study**	**200**	**South Indian**	**35.96 **± **3.75**	**30.38 **± 2.84	**124.87 ± 10.49**	**799.29 ± 134.05**

**Table 9:** Comparison of foramen magnum variables in female CT scans with previous studies.

**Authors name **	**Sample size**	**Target population**	**APD**	**TD**	**Circumference**	**Area **
Murshed et al. [2] 2003	110	Turkish population	34.6 ± 3.16	29.3 ± 2.19	-	795.0 ± 99.32
Garcia et al. [5] 2011	100	South American	31.9 ± 2.6	30.1 ± 2.4	-	798 ± 115
Uthman et al. [6] 2012	88	Iraqi population	32.9 ± 2	27.3 ± 2.2	92.6 ± 6.5	670.2 ± 93.7
Erdil et al. [7] 2010	54	Turkish population	34.41 ± 3.89	29.98 ± 2.78	-	-
Uysal et al. [8] 2005	100	Turkish population	34.87 ± 2.63	28.93 ± 2.43	-	-
**Present study**	**200**	**South Indian **	**33.83 **± **3.51**	**27.98 **± 2.53	**116.60 ± 9.67**	**697.77 ± 116.63**

## Data Availability

The data used to support the findings of this study are included within the article.
